# Supplementary material for: Institutionalising urogenital schistosomiasis surveillance: Best practices to improve female genital and urinary schistosomiasis control in South Africa
Source: PLoS Negl Trop Dis. 2025 Jun 4;19(6):e0012640. doi: 10.1371/journal.pntd.0012640 (PMC12164197; doi:10.1371/journal.pntd.0012640)
Supplement: Attachment — Submitted filename: Response to Reviewers.docx [file pntd.0012640.s005.docx]

**Response to Reviewer’s comments**

Thank you to all the reviewers. The following Table highlights all the issues that were addressed.

| **Examiner** | **Manuscript section** | **Issue** | **How it is addressed** | **Line number in the revised manuscript** |
| --- | --- | --- | --- | --- |
| **Examiner 1** | **Method** | 1. **Line 195**: The rationale behind the study would benefit from further elaboration on the scope of the control measures considered, particularly regarding deworming efforts for adolescent girls and women of reproductive age (see WHO Policy Brief). What was the reason for omitting this aspect from your study’s focus? | The sentence was revised to make it clear that all the at-risk community members would benefit from the mass drug administration. | Lines 225 - 227 |
|  |  | 2. **Address as limitations**: a. **Data Analysis**: There was no triangulation of data. Stakeholder interviews were not conducted to gain further insight into the process, but this is addressed in the limitation chapter. b. The authors have utilised descriptive analysis; please mention this as an limitation into the limitations chapter of your study. | A sentence was added under study limitation | Lines 491 - 492 |
|  | **Results** | 1. **Revision of Table 2**: I recommend adding a holistic and sustainable approach to FGS control to the list of gaps, emphasizing the need for greater coordination between the NTD and SRH sectors. Several articles can support this claim. Below are a few for your consideration. •Engels D, Hotez PJ, Ducker C, Gyapong M, Bustinduy AL, Secor WE, Harrison W, Theobald S, Thomson R, Gamba V, Masong MC. Integration of prevention and control measures for female genital schistosomiasis, HIV and cervical cancer. Bulletin of the World Health Organization. 2020 Sep 9;98(9):615. •Williams CR, Seunik M, Meier BM. Human rights as a framework for eliminating female genital schistosomiasis. PLOS Neglected Tropical Diseases. 2022 Mar 3;16(3):e0010165. •Pillay LN, Umbelino-Walker I, Schlosser D, Kalume C, Karuga R. Minimum service package for the integration of Female Genital Schistosomiasis into sexual and reproductive health and rights interventions. Frontiers in Tropical Diseases. 2024 Jun 17;5:1321069. | This has been added in Table 2 to include the lack of coordination between NTD and sexual reproductive health sectors. The references have also been updated. | Line 281 (point number 6)  Lines 675 - 683 |
|  |  | 2.**Improve Figures 4 and 5**. Currently, both absolute values (number of cases) and proportions (percentages) are presented on the same axis, making it difficult to understand. Consider using separate y-axes for the absolute numbers and the percentages, or improve the labelling of the axes for better clarity. | Figure 4: This has been revised and the percentages were removed to avoid confusing.  Figure 5 was doublechecked and the percentage is clearly indicated on the secondary axis and the number of cases primary axis | Line 342  Line 351 |
|  | **Conclusion** | Reviewer #1: **Major Revision** Your discussion provides a solid foundation for improving the management of schistosomiasis and FGS in South Africa. Please consider the following suggestions:  1. I strongly recommend adding a paragraph that discusses the potential benefits of integrating schistosomiasis and FGS surveillance into existing sexual and reproductive health and rights (SRHR) programs. This addition would present a more comprehensive approach to addressing both schistosomiasis and FGS in South Africa and beyond. | A sentence has been added after a sentence that was talking about integration of NTDs with the HIV, STI, and reproductive health programs. | Lines 374 - 384  Lines 469 - 476 |
|  |  | 2. Include a discussion on aligning national policies with international frameworks regarding SRHR and NTDs. | This has been addressed | Lines 471 - 476 |
|  |  | 3. Consider including a comparison with other countries that have institutionalized urogenital schistosomiasis surveillance, highlighting what lessons can be learned from their experiences. | This has been addressed | Lines 379 - 384 |
|  |  | 4. Address the limitations of the descriptive analysis in your discussion. | This has been addressed under study limitation paragraph. | Lines 491 - 492 |
|  |  | 5. Add a conclusion that summarizes specific recommendations for policymakers, international organizations, and local stakeholders to support the institutionalization of urogenital schistosomiasis surveillance. | A paragraph has been added to address this. | Lines 374 – 384  Other recommendations:  Lines 397 – 399  Lines 407 – 412  Lines 416 - 419 |
|  | **Editorial and Data Presentation Modifications?** | Reviewer #1: **Minor Revision**  The document uses various fonts and font sizes. Please standardise them to ensure uniformity throughout. | This has been doublechecked throughout the manuscript. | Line 216 font formatted. |
|  | **Summary and General Comments** | Reviewer #1: **Minor Revision** The paper is well-organized, with a clear and logical structure that effectively guides the reader through the research process. The writing is coherent and concise, making complex ideas accessible without sacrificing depth or detail. The paper highlights critical gaps in surveillance, treatment, and intersectoral collaboration, and make a strong case for improving the existing systems. Minor revisions are needed. Please consider expanding through the paper on the need for integration surveillance into SRHR programs.  *Introduction* 1.The introduction is comprehensive and provides a solid foundation for the study by addressing the burden, challenges, and gaps in schistosomiasis control in South Africa. However, it would be further enhanced by adding a paragraph discussing the potential for integrating schistosomiasis surveillance into Sexual and Reproductive Health (SRH) programs. Additionally, a brief reference to the recently published WHO policy brief titled "Deworming adolescent girls and women of reproductive age. Policy brief" could strengthen the rationale for such integration and highlight its relevance to addressing FGS and advancing public health strategies. | The integration with SRHR and surveillance has been addressed, and WHO reference used. | Lines 374 – 384  Lines 471 - 476 |
|  |  | 2.Please consider changing the term “urinary schistosomiasis” and “schistosomiasis” to "urogenital schistosomiasis”. It is more appropriate since your manuscript works with the intersection of FGS, an infection in both the urinary and genital organs. I suggest also updating the legend and caption in figures and tables. | This has been addressed were relevant. | Throughout the manuscript |
|  |  |  |  |  |
| **Examiner 2** | **Method** | Reviewer #2: Objectives and Hypothesis:  The manuscript outlines the objectives but lacks a clear, testable hypothesis.  Suggestion: Explicitly state a hypothesis related to the integration of FGS into existing surveillance systems and expected outcomes. | A sentence has been added under methods to address this issue. | Lines 230 - 232 |
|  |  | Study Design Appropriateness:  The descriptive study design is appropriate for analyzing surveillance data, but there is no control or comparison group.  Suggestion: Justify the choice of a descriptive study design and discuss how it limits or strengthens the conclusions. | This has been addressed under methods and study limitations. | Lines 158 – 160  Lines 491 - 492 |
|  |  | Population Description:  The study examines surveillance data from public health facilities but does not fully describe the demographics of the affected population.  Suggestion: Provide more details on the geographic, demographic, and socioeconomic characteristics of the population covered by the surveillance systems. | This has been clarified under methodology, and the results section is the one that outlines the populations that are mostly affected. | Lines 154 - 157 |
|  |  | Sample Size and Power Analysis:  The manuscript does not discuss sample size calculations or statistical power.  Suggestion: Justify whether the number of cases reported is sufficient for meaningful analysis, and discuss potential biases due to underreporting. | There was no sample size calculation because the focus was on describing what currently exists in the country. The underreporting has been mentioned as a limitation to the study. | Lines 487 - 489 |
|  |  | Statistical Analysis:  The manuscript mainly relies on descriptive statistics without more advanced statistical testing.  Suggestion: If feasible, incorporate statistical comparisons (e.g., chi-square tests for differences in reporting rates between provinces or health sectors) to strengthen conclusions. | This would have been very useful. However, it may not be possible because the disease is neglected, there is possibly huge underreporting, and there is no tracking of reporting in the country (there is also no zero reporting). The study findings and recommendations would help the country address these challenges. |  |
|  |  | Data Sources and Collection: Ensure clarity on how data were extracted, cleaned, and analyzed, including potential limitations in data accuracy. | This has been clarified under methodology. Unfortunately, the NHLS, NDoH and NICD extracted the data themselves and it was only shared in excel spreadsheet after extraction. | Line 196 |
|  |  | Ethical Considerations: While ethical approvals are noted, discuss how patient confidentiality and data protection were ensured. | Patient names and national identification was not included in the excel spread sheets, except for data from NICD (the NMC data) and this was removed during analysis and will not be shared. The variables that were included are specified in the methods. | Lines 198 – 201  Lines 205 – 214  Line 216 |
|  |  | Potential Biases: Address limitations such as reporting inconsistencies across surveillance systems and how they might affect findings. | This has been addressed under study limitations. | Lines 487 - 489 |
|  | **Results** | Reviewer #2: The results align with the stated objectives and methodology but could benefit from more explicit connections to the research questions.  Suggestion: Clearly reference the analysis plan in the results section to demonstrate how findings support the study's aims. | This has been addressed under revised discussion section. | Lines 230 – 232  From Line 368 |
|  |  | The results are well-structured and include key findings from multiple data sources. However, some sections could provide additional interpretation of the trends observed.  Suggestion: Expand on the implications of the data trends, particularly the declining case reports during the COVID-19 pandemic and the gender disparity in schistosomiasis prevalence. | This has been addressed under discussion. | Lines 456 - 461 |
|  |  | The figures and tables are informative, but some require clearer labeling and improved consistency in formatting.  Suggestion: Ensure uniformity in table structures and provide concise yet informative figure captions. | This has been addressed. | Lines 281 - 360 |
|  | **Conclusion** | Reviewer #2: The conclusions are generally aligned with the findings but could be strengthened by explicitly linking key results to recommendations.  Suggestion: Clearly restate the most significant findings and how they support the proposed recommendations. | This has been addressed. | From Lines 368 (revised discussion and conclusion section) |
|  |  | The manuscript acknowledges some limitations, such as inconsistent reporting, but does not fully explore their impact on the findings.  Suggestion: Expand on how limitations may affect the interpretation of results and suggest ways to address them in future research. | This has been addressed under discussion. | Lines 487 – 489  Lines 491 - 492 |
|  |  | The discussion highlights the need for improved FGS surveillance, but it lacks a detailed explanation of how the findings contribute to broader knowledge.  Suggestion: Provide a clearer discussion on how this study builds on previous research and offers new insights into schistosomiasis control. | This has been addressed under discussion. | Lines 370 – 384  Lines 400 - 412 |
|  |  | The manuscript emphasizes the importance of integrating FGS surveillance into existing health systems, which is a critical public health issue.  Suggestion: Strengthen the discussion on the policy implications of these findings, particularly in the context of neglected tropical disease control strategies. Authors should elaborate on practical steps for integrating FGS surveillance into existing systems. | This has been addressed under discussion and reference made to WHO and a paper that clearly recommends how integration should be done. | Lines 370 – 384  Lines 400 – 412  Lines 471 - 476 |
|  | **Editorial and Data Presentation Modifications?** | Reviewer #2: Abstract: Consider briefly mentioning that FGS is currently not a notifiable medical condition and how this impacts surveillance. | This had been included but it has been expanded. | Line 61 |
|  |  | Figures & Tables: Ensure consistency in the way data are reported across figures and tables for clarity. | This has been addressed. | Lines 281 - 360 |
|  |  | Grammar & Style: Some sentences are complex and lengthy; consider breaking them into shorter, clearer statements for better readability. | This has been addressed by breaking sentences into more than one and avoiding losing the meaning, especially under discussion. | From Lines 368 (revised discussion and conclusion section) |
|  |  | The writing is dense and technical, making it harder for non-specialists to follow. Some sections (e.g., Discussion) are repetitive, reiterating findings without additional interpretation.  Suggestion: Improve conciseness and readability, especially in the Discussion. Use bullet points or summarizing statements where appropriate. | This has been addressed by breaking sentences into more than one, especially under discussion. | From Lines 368 (revised discussion and conclusion section) |
|  | **Summary and General Comments** | Reviewer #2: This manuscript presents a valuable analysis of existing schistosomiasis surveillance systems in South Africa, highlighting the lack of institutionalized surveillance for female genital schistosomiasis (FGS). The study is well-structured and informative, with clear objectives and a thorough review of available data. However, there are areas that require further clarification and refinement to enhance its impact and clarity. Addressing the points above will improve its clarity, impact, and practical relevance for policymakers and researchers.  We appreciate the authors' valuable work and encourage revisions to strengthen the manuscript further. |  |  |
|  |  | Reviewer #2: This manuscript presents a valuable analysis of existing schistosomiasis surveillance systems in South Africa, highlighting the lack of institutionalized surveillance for female genital schistosomiasis (FGS). The study is well-structured and informative, with clear objectives and a thorough review of available data. However, there are areas that require further clarification and refinement to enhance its impact and clarity. Addressing the points above will improve its clarity, impact, and practical relevance for policymakers and researchers.  We appreciate the authors' valuable work and encourage revisions to strengthen the manuscript further. |  |  |
